# Altered Mitochondrial Signalling and Metabolism in Cancer

**DOI:** 10.3389/fonc.2017.00043

**Published:** 2017-03-20

**Authors:** Esita Chattopadhyay, Bidyut Roy

**Affiliations:** ^1^Human Genetics Unit, Indian Statistical Institute, Kolkata, India

**Keywords:** mitochondria, cancer, metabolism, bioenergetics, hypoxia

## Abstract

Mitochondria being the central organelle for metabolism and other cell signalling pathways have remained the topic of interest to tumour biologists. In spite of the wide acceptance of Warburg’s hypothesis, role of mitochondrial metabolism in cancer is still unclear. Uncontrolled growth and proliferation, hallmarks of tumour cells, are maintained when the cells adapt to metabolic reprogramming with the help of altered metabolism of mitochondria. This review has focussed on different aspects of mitochondrial metabolism and inter-related signalling pathways which have been found to be modified in cancer.

## Introduction

**Figure 1:**
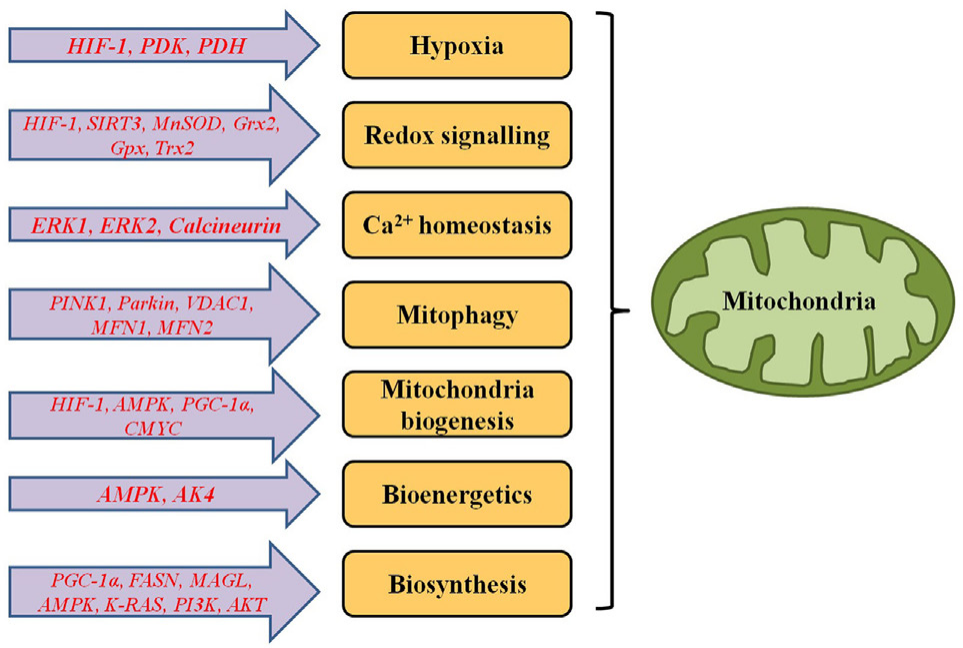
**Genes responsible for functional alterations in metabolic and signalling pathways of mitochondria in cancer cells**.

Production of major amount of energy for the cell by oxidative phosphorylation is the most essential function of mitochondria. Other mitochondrial functions include apoptosis or programmed cell death, Ca^2+^ homoeostasis, etc. It has its own circular genome [mitochondrial DNA (mtDNA)], which codes for protein subunits for oxidative phosphorylation, tRNAs, and rRNAs. Some of the proteins involved in mitochondrial structure and functions are encoded by nuclear genome. Mutations in mtDNA are being studied and found to be causal for different mitochondrial diseases including cancer (1, 2). Implications of mitochondrial function in cancer seem to be well-debated question. Manifestation of cancer includes uncontrollable cell proliferation, inhibited cell death, angiogenesis, invasion into other tissues, etc. Proper functioning of mitochondria is required to maintain rapid growth and proliferation of cancer cells since tumour cell devoid of mitochondria grows very slowly (3–5). On the other hand, functional impairment of mitochondria is a common phenomenon in cancer cells since they undergo certain changes in metabolic pathways for their survival and maintenance (Figure 1). This review highlights metabolic reprogramming in cancer cell due to altered mitochondrial signalling.

## Hypoxia and Mitochondria

Hypoxia or oxygen deprivation is one of the key features of solid tumours and plays a significant role in different cellular functions including cell proliferation, survival, angiogenesis, metabolism, tumour evasion, and metastasis (6). It also regulates tumour cells to have reduced response to radiotherapy, resistance to chemotherapy and lower pH than normal cells (7–9). Proliferation rate of tumour cells is higher than the growth rate of new blood vessel formation, so newly generated cells are supplied with lower amount of oxygen. Depending on the aggressiveness of hypoxia, tumour cells either undergo apoptosis or adapt to the low oxygen environment and survive (10). The key coordinator of cellular mechanisms to adapt and survive in hypoxic condition is hypoxia-inducible factor 1 (*HIF-1*). It is a transcription factor which consists of two subunits: *HIF-1α* whose expression is regulated by abundance of oxygen and *HIF-1β* which is constitutively expressed. Limited oxygen availability induces expression of *HIF-1* which regulates expression of several other genes (11) functionally involved in the pathways of angiogenesis, cell death/survival, metabolism, pH regulation, cell adhesion, extracellular matrix remodelling, cell migration, and metastasis (9).

**Figure 2:**
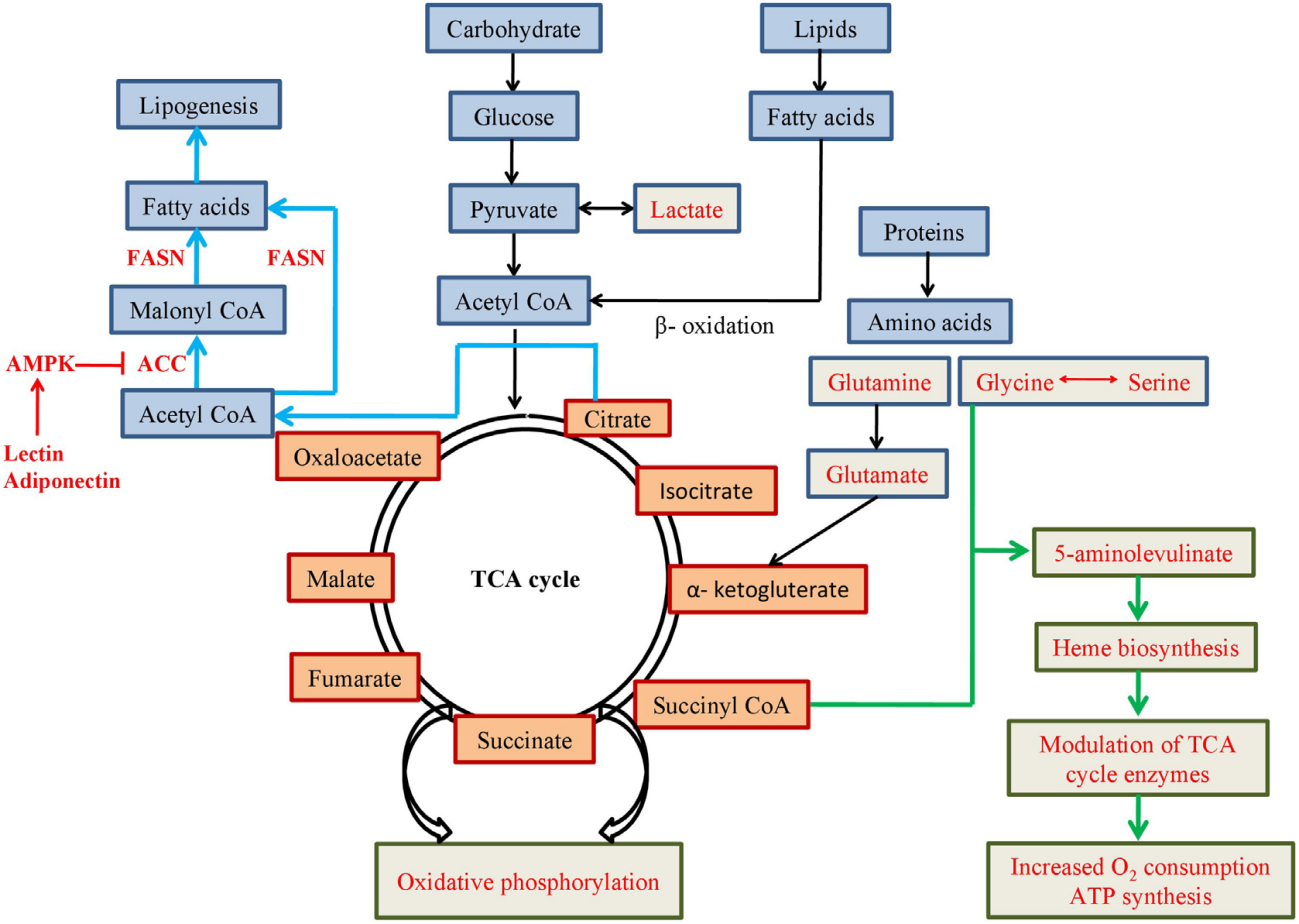
**Schematic representation of metabolic pathways involving mitochondrial metabolites**. Pathways or use of metabolites mentioned in red font are favoured in cancer cells for altered metabolism.

In presence of low oxygen in cell, pyruvate is mostly converted to lactate instead of acetyl CoA (Figure 2) and *HIF-1* induces expression of genes involved in the glycolytic pathway (such as glucose transporter, glycolytic enzymes, etc). *HIF-1* increases expression of pyruvate dehydrogenase kinase 1, a subunit of *PDK*, which blocks function of pyruvate dehydrogenase (*PDH*) enzyme leading to increase production of lactate (12). Increased amount of lactate also induces *HIF-1*, which not only blocks acetyl-CoA metabolism in mitochondria, but also reduces mitochondrial biogenesis as well as oxygen consumption (13, 14).

Although mitochondrial energy production is a more efficient method than that of glycolytic pathway, tumour cells perform glycolytic and other/altered metabolic pathways to generate energy. Mitochondrial electron transport chain (*ETC*) is involved in oxygen sensing since *ETC* consume most of the cellular oxygen. Certain inhibitors of *ETC* can block stabilisation of *HIF-1α* in hypoxic conditions, which signifies that functioning of *ETC* is required for hypoxia-mediated activities of *HIF-1*. During hypoxia, *ETC* complex III can release reactive oxygen species (*ROS*) into mitochondrial inter-membrane space and subsequently into the cytosol. Thus, mitochondrial *ROS* generation contributes to *HIF-1*α stabilisation under hypoxic condition of the cell (15).

## Mitochondrial Metabolism and Tumour Growth

Mitochondria coordinate anabolic as well as catabolic reactions combined with energy production to achieve the needs of cellular bioenergetics and biosynthesis. Acetyl CoA, the key ingredient of mitochondrial metabolism and energy production is generated from breakdown of glucose, amino acids, and fatty acids. Necessity of mitochondrial metabolism for tumour growth is a well-discussed but yet it is an inconclusive area of research. According to one hypothesis, rapid proliferation and growth of tumour cells require functional mitochondria as it is the major source of energy as well as supplier of metabolic building blocks for tumour cells. Anaplerotic pathways which maintain pools of metabolic intermediates for repeated usage of rapid growth and proliferation are well-adapted in cancer cells. TCA cycle intermediates are also utilised as carbon sources, such as production of α-ketoglutarate (α-KG) from glutamine by glutaminolysis, oxaloacetate production from pyruvate by pyruvate carboxylation, oxidation of branched chain amino acids, etc., to the anaplerotic activity of cancer cells (16–18).

Perturbation of cell signalling pathways, such as *K-Ras, PI3K–Akt–mTROC1, Myc* signalling, play significant roles in mitochondrial metabolism of cancer cells. *K-Ras* oncogene decouples glucose and glutamine metabolism. It exhibits enhanced glycolytic activity as well as increased usage of glutamine as a carbon source of TCA cycle (19). *PI3K–Akt* signalling pathway is found to be altered very frequently in different cancers. *mTORC1*, being one of the major targets of activated *Akt*, regulates growth factor signalling, energy state, and nutrient and oxygen availability in cancer cells (20–23). Oncogenic activation of *Myc* leads to activation of genes involved in glycolysis, glutamine metabolism, and mitochondrial biogenesis (24–27).

Mitochondrial TCA cycle enzymes, such as succinate dehydrogenase (SDH) and fumarate hydratase (FH), could function as mitochondrial tumour suppressors (28). Individuals with germline loss-of-function mutations in *FH* gene are predisposed to hereditary paraganglioma, pheochromocytoma, leiomyomatosis, and renal carcinoma (29, 30). Mutation-induced inactivation of SDH and FH results in accumulation of succinate and fumarate, respectively. These two metabolites leak out to cytosol to inhibit prolyl hydroxylase enzymes, which can promote cellular resistance to apoptotic signals or can activate pseudohypoxic response for triggering *HIF-1*-mediated glycolysis in cancer (31, 32). Elevated succinate and fumarate can also lead to consequent alteration of genome-wide histone and DNA methylation by inhibiting α-KG dependent dioxygenase (such as histone demethylases and TET family of 5-methylcytosine hydroxylases) activity (30, 33).

### Bioenergetics and Altered Metabolism

Warburg’s hypothesis (1956) suggested cancer cells exhibit increased glycolysis and lactate production irrespective of the presence of oxygen (34). Loss of tumour suppressors, activation of oncogenes, upregulation of *PI3K* pathway, and altered expression of mitochondrial metabolic enzymes may also result in increased glycolysis (35, 36). Rate of glycolysis is increased in cancer cells but glycolytic products may enter mitochondria at different points of TCA cycle for energy production and growth, migration and metastasis of cancer cells (37). On the other hand, cancer cells with mitochondrial respiration defects rely on energy generated by glycolysis. Inhibiting glycolysis in these cancers results in depletion in ATP production and finally leads to apoptosis of the cells (35, 38). But in general, mitochondrial metabolism is necessary for cancer cell survival, proliferation, and growth. So, cancer cells adopt multiple mechanisms to maintain proper functioning of mitochondria. Moreover, in hypoxia and nutrient deficient conditions, mitochondria can modify its energy production as well as utilisation to adapt to the tumour microenvironment since mitochondrial ETC can produce ATP even at very low oxygen level (39, 40). In these conditions, cancer cells can adapt different mechanisms to maintain their ATP/ADP ratio and decrease their demand for ATP as well as cellular functions which are ATP-dependent. In lower energy state (higher ADP/ATP or AMP/ATP ratio), activation of AMP kinase (*AMPK*) is triggered by mitochondrial adenylate kinase (*AK4*) and activates catabolic pathways (such as fatty acid oxidation) to stimulate ATP production (41, 42).

### Biosynthesis of Metabolites

Rapid cell division and growth of cancer cells require good supply of macromolecules. Various anabolic pathways utilise simpler and smaller nutrient molecules such as glucose, fatty acids, and amino acids to produce larger molecules or building blocks for the cells. Mitochondria which work as a central organelle of cellular metabolic pathways perform different anabolic reactions to generate intermediate products for macromolecules.

Biosynthetic pathways of fatty acids and amino acids were found to be upregulated in cancer cells indicating their importance as metabolites (43, 44). Except pyruvate from glycolysis, other metabolic substrates from amino acid and fatty acid metabolism are also transported to mitochondria for further metabolic activities. When pyruvate is mostly being used for lactate production, the metabolites from fatty acid and amino acid metabolism pathways play central role for providing metabolic substrates to mitochondria. In hypoxic condition, when acetyl CoA production from pyruvate is impaired, glutamine acts as a biosynthetic precursor of acetyl CoA for sustainability of the tumour (40).

#### Glucose

Glucose is the most widely used nutrient in the body. Uptake of fluorodeoxyglucose (Fl-18), a glucose analogue, measured by positron emission tomography in cancer cells, was found to be increased as the cancer progresses (45, 46). Recent evidences indicate, although lactate production is induced in cancer cells, glucose also produces pyruvate which enters into mitochondrial TCA cycle. Pyruvate is metabolized into *acetyl CoA* by mitochondrial *PDH* complex and it is then converted to citrate by citrate synthase. Citrate is either converted to isocitrate within TCA cycle or is transported to the cytosol to yield cytosolic *acetyl CoA* which is used as a substrate for lipogenesis and acetylation.

The final and rate limiting factor of glycolytic pathway is the M2 isoform of pyruvate kinase (*PKM2*). *PKM2* dimer has low kinase activity and drives pyruvate to lactate formation, whereas *PKM2* tetramer which has higher kinase activity promotes the pyruvate to enter mitochondria for ATP production *via* oxidative phosphorylation pathway (47). *PKM2* is commonly found to be highly expressed in cancers and induces rate of glycolysis, cell proliferation, migration, and invasion (48–50). It functions as a coactivator of *HIF-1* by enhancing the Warburg effect in cancer. *PKM2* can also regulate cancer progression by activating mTOR or EGFR signalling pathway (47, 51, 52).

#### Amino Acids

Besides glucose, amino acids are also important substrates in mitochondrial biosynthesis of lipid and protein molecules. In absence of glucose, glutamine acts as major nutrient in cancer cells. Importance of glutamine in cellular metabolism is due to its ability to donate its carbon and nitrogen into different growth promoting pathways. Although, glutamine is a non-essential amino acid, during rapid growth and cell proliferation in tumour, it is required to be imported from outside source to meet the high demands of it. Glutamine catabolism occurs inside mitochondria by glutaminase to produce glutamate and ammonia. Glutamate functions as a precursor of cellular antioxidant glutathione and donates amino groups to synthesise non-essential amino acids like glycine, alanine, aspartate, and serine. Glutamate is also converted to α-KG, enters mitochondrial TCA cycle to provide carbon skeleton for macromolecules and contribute in ATP synthesis by oxidative phosphorylation (53). Requirement of glutamine can be variable in different tumours. In some tumours, glutamine plays as an essential amino acid, whereas some tumours seem to be independent of glutamine and utilise glucose-derived pyruvate as major source of nutrient (54).

Glycine and serine are two other amino acids which have been identified to have significant importance in cancer metabolism. They are inter-convertible and biosynthesis of both the amino acids comprises cytosolic as well as mitochondrial enzymatic pathways. Hyper-activation of serine and glycine biosynthesis can accelerate tumourigenesis (55). Activation of mitochondrial, but not cytosolic, enzymes for glycine biosynthesis, which is known to be significantly correlated with cancer cell proliferation, implicates importance of mitochondria in cell proliferation in cancer (56). Moreover, glycine and succinyl CoA might condense to form 5-aminolevulinate which is key precursor for haem biosynthesis in mitochondria (57). Iron modulates expression of four enzymes of TCA cycle, aconitase, citrate synthase, isocitrate dehydrogenase, and SDH. It also reduces glucose utilisation by increasing oxygen consumption and ATP synthesis in mitochondria. When iron is depleted, glycolysis and lactate formation are significantly increased to compensate for ATP production in mitochondria (58). Increased supply and generation of *haem* induces oxygen consumption and energy production in mitochondria for progression of cancer cells, such as lung cancer (59).

Mutations in isocitrate dehydrogenase enzymes *IDH1* (cytosolic) and *IDH2* (mitochondrial) are highly frequent in glioma and AML, though rare in other cancers. Recurrent mutations in *Arg132* in *IDH1* and *Arg140* and *Arg172* in *IDH2* gene comprise 90% of the total mutations (60–63). Mutant *IDH1 or IDH2* produces 2-hydroxyglutarate (2-HG), instead of α-KG (64, 65), which functions as an oncometabolite. Accumulation of 2-HG in cells results in epigenetic dysregulation followed by aberrant gene expression (62). *IDH1* and *IDH2* mutations are also responsible for hypermethylation, decreased differentiation, and increased stemness of cancer cells as well as *HIF-1*α-mediated angiogenesis and growth of tumour (66–68).

#### Fatty Acids

Lipogenesis is a common feature of different cancers. Although normal cells mostly depend on exogenous sources of lipid, cancer cells can execute *de novo* synthesis of fatty acids. It is one of the major components of cellular lipid which is needed for cell membrane synthesis, energy production, lipid modification of proteins, and signalling molecule production in highly proliferative cancer cells. In most of the cancers, cause of increased lipogenesis is due to overproduction of enzyme, fatty acid synthase (*FASN*). It performs the final catalytic step to convert acetyl CoA and malonyl CoA to fatty acids and functions as an oncogene by promoting cancer cell proliferation and growth. Suppression of *FASN* results in cell cycle arrest, reduction in cancer cell proliferation, and increase in apoptosis (69–74). Another enzyme monoacylglycerol lipase that controls monoacylglycerol levels in normal cells is highly expressed in aggressive cancers and regulates a fatty acid network. This network has lipids with oncogenic potential and increases pathogenicity by promoting invasion, survival, and growth of the tumour (44). Alternatively, low glucose content or food deprivation has been found to increase fatty acid oxidation in cancer (75). Fatty acids are considered major source of energy in healthy cells as they can provide twice ATP than carbohydrates. Fatty acid oxidation (also known as β-oxidation) promotes cell proliferation and inhibits apoptosis in tumour cells (76, 77). Other than ATP production, β-oxidation-derived NADPH is the key mediator of oxidative stress as well as the coenzyme for anabolic reactions (78). *PPAR* genes play essential roles in β-oxidation by modulating enzymes involved in the pathway (79). PPAR-gamma coactivator-1α (*PGC-1α*) is activated by low glucose content and activates *SIRT1*. Both *SIRT1* and *Sirtuin-3* (*SIRT3*) activate metabolic enzymes for β-oxidation of fatty acids into acetyl CoA (80, 81) in mitochondria. Acetyl-CoA carboxylase (*ACC*) converts acetyl CoA to malonyl CoA in fatty acid synthetic pathway and is suppressed by *AMPK*. Hormones, such as leptin and adiponectin, activate *AMPK* and suppress *ACC* to inhibit fatty acid synthesis and increase fatty acid oxidation (82, 83).

#### Acetate

Recent studies on cancer metabolism have identified that certain tumours use exogenous acetate as an alternative source for producing acetyl CoA (84–86). Even in a glucose abundant scenario, glucose and acetate are simultaneously used to produce TCA cycle intermediates. Tumours, especially those with hypoxic conditions can produce 50% of their acetyl CoA from acetate, while the remaining amount is produced by utilising glucose and glutamine as carbon sources (87). Disease progression is correlated to the activity of the nucleo-cytosolic acetyl-CoA synthetase (*ACSS2*) (88). *ACSS2* uses acetate as a carbon source to generate acetyl CoA. Tumours which utilise acetate as a carbon source also show elevated activity of *ACSS2*. Mutations causing *PTEN* and *BRAF* inactivation driving AKT and ERK pathways have been found to play role in increased expression of *ACSS2*. Tumours devoid of this enzyme consume less amount of acetate, undergo cell death, and subsequently reach to a reduced tumour size (86).

#### Mevalonate–Isoprenoid Pathway

Mevalonate–isoprenoid pathway is used for cellular cholesterol biosynthesis and protein prenylation and hyper-activated in many cancers. It activates proteins of RAS signalling pathway by prenylation leading to cell transformation and malignancy (89, 90). Activation of this pathway shows chemoresistance in cancer cell line (91) and also induces cancer metastasis by epithelial mesenchymal transition, remodelling of cytoskeleton, cell motility and cell polarity (non-canonical *Wnt*/planar pathway) (92). Multiple factors can regulate mevalonate pathway. Mutant form of tumour suppressor protein *P53* induces protein prenylation of mevalonate pathway, thus maintaining malignancy, three-dimensional growth of tumour, and invasive growth (90, 93, 94). Activation of *ARID1A* (a subunit of *SWI/SNF* chromatin remodelling complex) and *IGF-1R/AKT/mTOR* axis result in increased activity of mevalonate pathway in ovarian and colorectal cancer, respectively (95, 96). *Kallikrein-related peptidase 5* (*KLK5*) protease can inhibit enzymes of mevalonate pathway in breast cancer (97). Drugs like statin, quinazoline, and simvastatin show anticancer effects by inhibiting mevalonate pathway, which indicates possibility of building new therapeutic strategies targetting this pathway (92, 98–103).

## Mitophagy

Mitophagy or selective degradation of damaged mitochondria is a quality control mechanism for maintaining homoeostasis of functional mitochondria in cell (104). Key regulation of mitophagy is executed through *PINK1–Parkin* pathway. Under stress conditions like hypoxia and nutrient deficiency, *Parkin (E3 ubiquitin ligase*) is recruited by *PINK1* for proteasomal degradation of target proteins (VDAC1, mitofusin 1, mitofusin 2) at mitochondrial outer membrane (105–108). Loss-of-function mutation, copy number variation, or deletion of *PARK2* (encoding Parkin protein) resulted in retaining damaged mitochondria in different cancers and, thus, indicates tumour suppressing role of mitophagy. In solid tumours, loss of Perkin induces aerobic glycolysis, thus supporting Warburg’s hypothesis (109).

## Redox Signalling

Reactive oxygen species are, mostly, by-products of electron transporting systems in mitochondria. Although ROS have some important roles in transcriptional activation, cell proliferation, and other signalling pathways but excess amount of ROS is responsible for damaging cellular DNA, lipids, and proteins (110). Antioxidant systems of the cell provide protection against excess amount of ROS. Excess redox signalling leads to carcinogenesis, tumour development and progression, cell migration, and angiogenesis. An increased quantity of ROS can activate hypoxia-mediated *HIF-1*α signalling pathway which might cause metabolic shift from oxidative phosphorylation to glycolysis by increasing expression of glycolytic enzymes. *HIF-1*α also reduces expression of tumour suppressor *SIRT3* which functions in activating antioxidants in mitochondria of healthy cell. Loss of *SIRT3* is reported in many cancers and it results in continuous steady state level of ROS and oxidative stress. Mitochondrial antioxidant systems which regulate ROS level are manganese superoxide dismutase (MnSOD or SOD2), mitochondrial glutaredoxin, glutathione peroxidise, and thioredoxin 2 (111–114). Antioxidants have dual role in redox signalling pathway. In physiological ROS signalling, they function as tumour suppressor by inhibiting ROS-induced cell proliferation and survival needed for cancer progression. By contrast, excess ROS in tumour microenvironment promotes apoptotic signals and, then, antioxidants suppress those signals and act as tumour promoters. But, generally, these antioxidant systems protect cells against oxidative stress and ROS-induced cell death.

## Mitochondria Biogenesis

Biogenesis of mitochondria is required in cells with high energy demands. DNA double strand breaks, induced by anticancer drugs, activate *ataxia telangiectasia mutated* to activate α subunit of *AMPK* to increase mitochondrial biogenesis (115). Contradicting Warburg’s hypothesis, recent studies have proposed a two-compartmental metabolic system in cancer (116, 117). Cancer cell and surrounding stromal cells undergo metabolically symbiotic relationship, where the cancer cells have active mitochondria and increased mitochondrial biogenesis but the stromal cells contain dysfunctional mitochondria and take up glycolytic pathway (117). Ketone bodies are also major energy sources for mitochondria and they are synthesized in tumour stroma with the help of enzymes, such as *HMGCS2, HMGCL, BDH1*, and re-utilised in tumour cells (116). Severe oxidative stress leads to apoptotic cell death, whereas mild oxidative stress can increase mitochondrial biogenesis as well as mtDNA content in cancer cells. Key regulator of mitochondrial biogenesis is *PGC-1*α, which regulates expression of nuclear genes for respiratory chain function, transcription, and replication of mtDNA by activating transcription factors (*NRF-1* and *NRF-2*), tumour suppressor genes (*SIRT3*), nuclear coded mitochondrial enzymes (*POLRMT*), and transcription factor (mtTFA) (25, 118–121). Activated *PGC-1*α in invasive cancer cells increases oxidative phosphorylation, oxygen consumption, and mitochondrial biogenesis and finally cell’s potential for distant metastasis. Activation of *PGC-1*α for mitochondrial biogenesis can occur through different signalling pathways (such as *AMPK, NO–cGMP, cAMP–PKA–CREB, p38*, and *ERR*α pathways) (122–124). Alternatively, *HIF-1* negatively regulates mitochondrial biogenesis and oxygen consumption by inhibiting *C-MYC via MXI-1* dependent and *MXI-1* independent pathways in renal carcinoma (14). Translation of nuclear-encoded mitochondrial function-related genes, protein folding, and entry in mitochondrial sub-compartments are regulated by *mTOR* which is often found to be hyper-activated in cancer (125–128). Suppression of *mTOR* results in damage and loss of mitochondria in cancer (129). *MYC*, which has known oncogenic effects in various cancers, induces nuclear-encoded mitochondrial gene expression and mitochondrial biogenesis in cancer (14, 25, 130). Sustained expression of *MYC* can lead to increased production of *ROS* and subsequent genomic instability and mitochondrial dysfunction (131, 132).

## Mitochondrial Fission and Fusion

Fission and fusion, which are the key components in mitochondrial dynamics, modulate mitochondrial morphology and subsequently regulate essential cellular mechanisms such as cell growth, cell division, and distribution of mitochondria during differentiation (133–135). Imbalance in expression of fission controlling protein dynamin-related protein 1 (*Drp1*) and fusion controlling protein *Mfn1* (mitofusin 1) is observed in different cancers. Increased fission or mitochondrial fragmentation due to high expression and activity of *Drp1* and decreased fusion due to loss of *Mfn1* activity are often linked to cancer cell migration, invasiveness, and metastasis (136–140). Pathways mediated by *p53, PINK1*, and mitochondrial membrane proteins are also found to be involved in regulation of mitochondrial fission as well as chemosensitivity of cancer cells (141–144). Other than proteins, mitochondrial lipids (cardiolipin, phosphatidylethanolamine, phosphatidic acid, diacylglycerol) also play important role in controlling mitochondrial dynamics (140).

## Calcium Homoeostasis

Calcium ion concentration is a key regulator of various signalling pathways of the cytosol and cellular organelles. Under physiological conditions, Ca^2+^ plays a beneficiary role by producing higher glycolytic and mitochondrial pathway enzymes (such as *PDH*, isocitrate dehydrogenase, α-KG dehydrogenase, ATP synthase, and α-glycerophosphate dehydrogenase), increasing oxidative phosphorylation activity and activating metabolite carriers (aspartate/glutamate carrier) of mitochondria. On the contrary, higher concentration of Ca^2+^ within mitochondria induces several negative effects on mitochondrial function which finally leads to apoptosis (145, 146). Mitochondrial stress (such as mtDNA depletion) in cancer cells results in increased cytosolic Ca^2+^, activation of calcium dependent *MAPK* (*ERK1* and *ERK2*) and calcineurin, increased anti-apoptotic proteins, and loss of pro-apoptotic proteins (147). Mitochondria-associated membrane (MAM) structure which is the interacting interface between ER and mitochondrial outer membrane, functions as the gateway of Ca^2+^ release from ER to mitochondria. Oncoproteins and tumour suppressor proteins residing on MAM control apoptosis *via* Ca^2+^ homoeostasis. Ca^2+^ release from ER as well as uptake by mitochondria are inhibited by several oncogenes like *AKT, Bcl2*, and *K-Ras* to trigger anti-apoptotic signalling in cancer cells (148–150). Functional loss of ER protein PERK and mitochondrial calcium channel (MCU) are also known to have anti-apoptotic effects in cancer (151–153).

## Cell Death

Cell death is a physiological regulator for development, tissue homoeostasis, stress, and also functions as tumour suppressor. Besides apoptosis, mitochondria are also found to be involved in other cell death mechanisms such as autophagy, necrosis, and necroptosis (programmed necrosis) (154–156). Proteins known as inhibitors of apoptosis (*IAP*s) are overexpressed in cancer and inhibit caspases or procaspases (primarily caspase-3 and caspase-7) to suppress apoptosis. Cancer cells with activated IAPs become highly resistant to radiation or chemotherapy (157). Anti-apoptotic proteins of *BCL2* family are overexpressed in cancer and inhibit the pro-apoptotic proteins to initiate the process of cell death. Thus, *BCL2* proteins are targetted by *BCL2*-inhibitors in cancer therapy to promote apoptosis (158, 159). Tumour suppressor, *P53*, plays an important role in promoting cell death. It is activated in ROS-dependent pathway and inhibits oncogenes *via* JNK-mediated signalling pathway leading to apoptosis in cancer (160, 161). Inhibited cell growth and increased apoptosis in cancer by P53 activation are also regulated by miRNA or SIRT2 dependent pathways (162, 163).

Mitochondrial fission- and fusion-related proteins, *Drp1* and mitofusin (*Mfn1* and *Mfn2*), are found to be involved in cell death (164). *Drp1* induces mitochondrial fragmentation and apoptosis in a *BAX/BAK*-mediated pathway. Overexpression of *Drp1* increases *ROS* production, release of cytochrome *c*, and *PARP* cleavage (165, 166). Being phosphorylated by *ERK, Mfn1* modulates apoptosis and fusion. Mutant *Mfn1* binds to *BAK* more strongly inducing *BAK* activation and cell death (167). *Mfn2* promotes anti-proliferative and pro-apoptotic effects *via PI3K–AKT* signalling pathway and lower expression of *Mfn2* is associated with poor survival in cancer (168, 169).

## Mutations in Mitochondrial Genome

Somatic mutations in mitochondrial genome (mtDNA) are common and frequently reported in different types of cancer (170–177). Functional consequences of these mutations are not well understood. These mutations are mostly point mutations, small insertion–deletions, or large scale deletions distributed in protein coding genes (177, 178). These mutations are thought to arise due to poor DNA repair mechanism and direct exposure to ROS, although oxidative stress is not always considered as a major contributor to somatic mutations (179). Mutations in coding genes might cause functional imbalance in respiratory chain. Mutant respiratory chain proteins promote elevation of ROS, tumour size, and glycolysis *via HIF-1*-mediated pathway in head and neck and prostate cancer (180, 181).

## Conclusion

Mitochondria are essential organelles for energy production but play important roles in carcinogenesis, cancer progression, and metastasis helping altered energy metabolism in cancer cells. Mitochondrial metabolism is also connected with other mitochondrial pathways such as redox signalling, Ca^2+^ signalling, mitophagy, and mitochondrial biogenesis. These pathways cross talk and seem to play important roles in cancer. Targeting mitochondrial pathways individually or in combination might be considered as future cancer therapy. Recently, cancer researchers are focussing on the metabolic reprogramming of cancer cells to use altered metabolites/oncometabolites for therapeutic approach.

## Author Contributions

EC wrote the draft of the manuscript and BR revised and finished it.

## Conflict of Interest Statement

Authors declare that the manuscript was written in absence of any commercial or financial relationships that could be constructed as a potential conflict of interest.

## Abbreviations

mtDNA, mitochondrial DNA; ETC, electron transport chain; ROS, reactive oxygen species.
